# Preclinical Assessment of a New Hybrid Compound C11 Efficacy on Neurogenesis and Cognitive Functions after Pilocarpine Induced Status Epilepticus in Mice

**DOI:** 10.3390/ijms22063240

**Published:** 2021-03-22

**Authors:** Marta Andres-Mach, Aleksandra Szewczyk, Mirosław Zagaja, Joanna Szala-Rycaj, Marta Kinga Lemieszek, Maciej Maj, Michał Abram, Krzysztof Kaminski

**Affiliations:** 1 Isobolographic Analysis Laboratory, Institute of Rural Health, Jaczewskiego 2, 20-090 Lublin, Poland; szewczyk.aleksandra@imw.lublin.pl (A.S.); lasius1981@wp.pl (M.Z.); szala-rycaj.joanna@imw.lublin.pl (J.S.-R.); 2 Department of Medical Biology, Institute of Rural Health, 20-090 Lublin, Poland; lemieszek.marta@imw.lublin.pl; 3 Department of Biopharmacy, Medical University of Lublin, Chodzki 4A, 20-090 Lublin, Poland; ewolucjonista@gmail.com; 4 Department of Medicinal Chemistry, Faculty of Pharmacy, Jagiellonian University Medical College, Medyczna 9, 30-688 Cracow, Poland; michal.abram@doctoral.uj.edu.pl (M.A.); k.kaminski@uj.edu.pl (K.K.)

**Keywords:** anticonvulsant, neuroprotection, neurogenesis, pilocarpine, antiepileptic drugs

## Abstract

Status epilepticus (SE) is a frequent medical emergency that can lead to a variety of neurological disorders, including cognitive impairment and abnormal neurogenesis. The aim of the presented study was the *in vitro* evaluation of potential neuroprotective properties of a new pyrrolidine-2,5-dione derivatives compound C11, as well as the *in vivo* assessment of the impact on the neurogenesis and cognitive functions of C11 and levetiracetam (LEV) after pilocarpine (PILO)-induced SE in mice. The *in vitro* results indicated a protective effect of C11 (500, 1000, and 2500 ng/mL) on astrocytes under trophic stress conditions in the MTT (3-[4,5-dimethylthiazole-2-yl]-2,5-diphenyltetrazolium bromide) test. The results obtained from the *in vivo* studies, where mice 72 h after PILO SE were treated with C11 (20 mg/kg) and LEV (10 mg/kg), indicated markedly beneficial effects of C11 on the improvement of the neurogenesis compared to the PILO control and PILO LEV mice. Moreover, this beneficial effect was reflected in the Morris Water Maze test evaluating the cognitive functions in mice. The *in vitro* confirmed protective effect of C11 on astrocytes, as well as the *in vivo* demonstrated beneficial impact on neurogenesis and cognitive functions, strongly indicate the need for further advanced molecular research on this compound to determine the exact neuroprotective mechanism of action of C11.

## 1. Introduction

Status epilepticus (SE) is defined as a serious medical condition characterized by continuous or rapidly recurring seizures without a recovery period between them. These states can have long-term consequences, including neuronal death, neuronal injury, and the alteration of neuronal networks [1]. The early phase after SE can lead to neurodegeneration, neuroinflammation, and abnormal neurogenesis in the hippocampus, although the extent of these changes depends on the severity and duration of seizures [2]. In many cases, permanent traumatic lesions caused by SE can lead to temporal epilepsy, very often refractory to treatment with a single antiepileptic drug (AED) [3]. This type of epilepsy usually requires the chronic use of at least two AEDs to stop or minimize seizures, which unfortunately carries the risk of side effects such as drowsiness or chronic fatigue, dizziness, or cognitive impairment. Problems with spatial orientation and learning and memory functions are associated with both the degenerative process of nerve cells and disorders of the process of neurogenesis [4]. Hippocampal neurogenesis is very sensitive to various physiological and pathological stimuli, including seizures, which have been proven to alter both the extent and the pattern of neurogenesis [5]. Moreover, it is thought that not only seizures but, also, antiepileptic treatment has a significant impact on neurogenesis. It is possible to find many publications based on *in vivo* and *in vitro* studies that present positive, as well as negative, influences of AEDs on the process of neurogenesis. Chen et al. [6], using topiramate (TPM) and lamotrigine (LTG), indicated the promotion of aberrant neuron regeneration by TPM, but not LTG, in the hippocampus after SE. Similar to LTG, levetiracetam (LEV) suppressed the development of spontaneous electroencephalography (EEG) seizures and aberrant neurogenesis following kainic acid (KA)-induced SE [7]. Valproic acid (VPA), a well-known anticonvulsant and mood stabilizer, reduced cell proliferation in the subgranular zone (SGZ) of dentate gyrus (DG) and impaired the ability of treated rats to successfully perform a hippocampus-dependent spatial memory test [8]. Additionally, it was shown that VPA induces abnormal visual avoidance and schooling behaviors in *Xenopus laevis* tadpoles [9]. The results from our investigations also confirm that long-term injection of VPA slightly decreased the total amount of newly born cells, while combination of VPA and arachidonyl-2’-chloroethylamide, a highly selective cannabinoid CB1 receptor agonist (ACEA), significantly increased the level of newborn neurons in the dentate SGZ in mice [10]. Juliandi and coworkers [11] showed that comparable postnatal cognitive functional impairment after prenatal VPA exposure in mice is caused by the untimely enhancement of embryonic neurogenesis, which leads to depletion of the neural precursor cells (NPCs) pool and, consequently, a decreased level of adult neurogenesis in the hippocampus. Similar results were obtained by Sakai et al. [12], indicating that prenatal VPA exposure in mice impairs neuronal migration in the adult dentate gyrus through the decreased expression of CXC motif chemokine receptor 4 (*Cxcr4)* in NPCs and, consequently, increases seizure susceptibility, whereas voluntary running overcomes these adverse effects. Sondosi and coworkers [13] showed that ethosuximide (ETS) has the ability to induce neuronal differentiation into GABAergic neurons that may explain one of the mechanisms of antiepileptic effects of ETS in rat forebrain stem cells.

In addition to intensively designed and synthesized chemical compounds with potential antiepileptic properties, a lot of attention is also paid to substances of natural origin, which are increasingly used in research with AEDs in various models of experimental epilepsy [10,14,15,16,17,18].

The results obtained by Kaminski and Obniska et al. [19,20,21] in a group of chemically diversified pyrrolidine-2,5-diones clearly showed anticonvulsant properties for several selected compounds in few animal models of epilepsy, including pilocarpine model of epilepsy (PILO). Looking for new potent multifunctional anticonvulsants with a broad spectrum of efficacy in the preclinical studies, we decided to combined on the common chemical template structural fragments of three AEDs active in three different animal models of epilepsy—namely, lacosamide (LCS), active in the maximal electroshock (MES) and six-hertz (6 Hz) seizure tests, etosuximide (ETS), effective in the pentylenetetrazol (PTZ) seizure test, and LEV, which acts potently in the 6-Hz seizure model. In consequence, on the basis of this assumption, we designed and synthesized a new compound, C11, with a hybrid structure that revealed a broad spectrum of activity in all aforementioned experimental seizure models [22].

Taking into consideration the anticonvulsant and neuroprotective activity of LEV in a mouse PILO model of epilepsy [23,24,25], the 6 Hz [26], and anticonvulsant activity of C11 in acute animal models of epilepsy [22], we decided to investigate an impact of the long-term treatment with C11 on neuroprotection, hippocampal neurogenesis, and cognitive functions in mice [27]. The results we obtained indicated that hybrid compound C11 used chronically has no negative impact on learning and memory functions in mice. Similarly, the long-term administration of C11 did not cause neuronal degeneration or neurogenesis in treated mice [27].

Considering the above-mentioned promising data, continuing research on compound C11, we decided to assess the potential neuroprotective properties of C11 *in vitro* and evaluate the impact of long-term treatment with C11 and LEV as a reference AED on neural stem cell proliferation, migration, and differentiation, as well as the cognitive functions and in the PILO model of status epilepticus (SE) in mice *in vivo*.

## 2. Results

### 2.1. Protective Abilities of C11—In Vitro Studies

**Figure 1:**
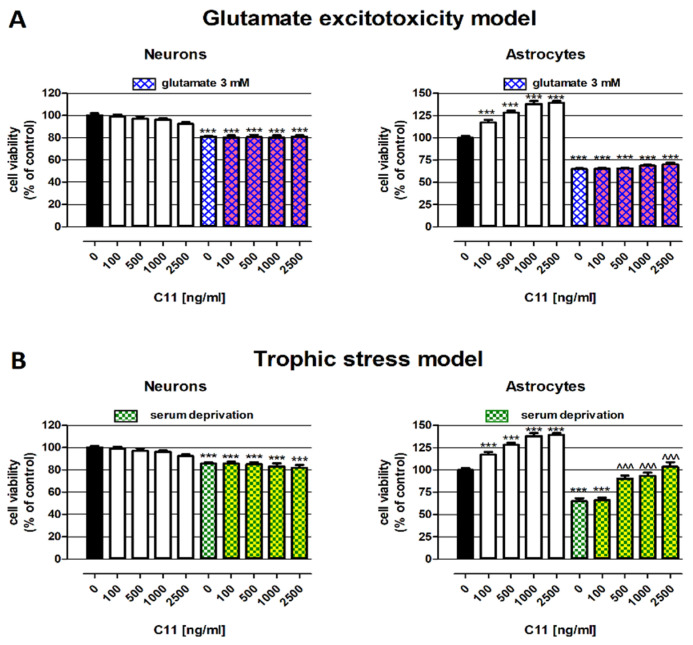
Impact of C11 on the viability of neurons and astrocytes under standard or degenerative conditions: (**A**) glutamate excitotoxicity and (**B**) trophic stress. Cells were exposed for 48 h to the investigated compound in concentrations ranging from 100 to 2500 ng/mL prepared in culture medium alone (control) or with 3-mM glutamate or serum-deprived cell culture medium. Cell viability (metabolic activity) was examined photometrically by the MTT assay. Results are presented as the mean ± SEM of 6–12 measurements. Statistically significant differences compared to the control (*black bar*) at *** *p* < 0.001. Statistically significant differences compared to the serum deprivation medium (white bar with pattern) at ^^^ *p* < 0.001. One-way ANOVA test Tukey’s post-hoc test.

In the first step, the C11 impact on neuron and astrocyte viability was performed using colorimetric MTT assay (cell metabolic activity examination) after 48 h of cell exposure to the tested compound in concentrations from 100 ng/mL to 2500 ng/mL dissolved in a culture medium suitable for the given cells. As presented in Figure 1 (left part of the graphs), C11 in the whole range of analyzed concentrations (from 100 to 2500 ng/mL) did not impact the neuron viability. On the contrary, C11, in a dose-dependent manner, increased the metabolic activity of astrocytes. The viability of astrocytes after treatment with C11 elevated from 117.1% of the control (100 ng/mL) to 139.1% of the control (2500 ng/mL).

In the next step, C11’s influence on the nerve cell viability under glutamate excitotoxicity conditions was tested. As presented in Figure 1A, 3-mM glutamate significantly decreased both the neuron and astrocyte viability by 19.1% and 35.1%, respectively. C11, in the whole range of analyzed concentrations, did not increase or weaken the negative effect of glutamate, which suggested a lack of protective properties against excitotoxicity evoked by glutamate.

In order to examine the influence of C11 on the nerve cell viability under trophic stress conditions, trophic factors were removed (supplement B27; neurons) from the standard cell culture medium, or its amounts were significantly reduced (fetal bovine serum (FBS); astrocytes). As presented in Figure 1B, the viability of the investigated nerve cells was lowered in response to trophic stress by 14.3% (neurons) and 35.1% (astrocytes). C11 administered to neurons in a culture medium without B27 was not able to reduce the negative impact of serum deprivation on the cell viability. On the contrary, C11 at concentrations 500, 1000, and 2500 ng/mL revealed a significant trophic effect in astrocytes. C11, at the mentioned concentrations, effectively protected astrocytes viability from inhibition caused by a 10-fold reduction of the serum amount in the culture medium.

### 2.2. Cognitive Functions and Neurogenesis—In Vivo Studies

#### 2.2.1. Evaluation of the Effects of Long-Term Administration of C11 and LEV on the Total Amount of Newborn Cells in the Dentate SGZ and Granular Cell Layer (GCL) of Mouse Hippocampus after PILO Induced SE

**Figure 2:**
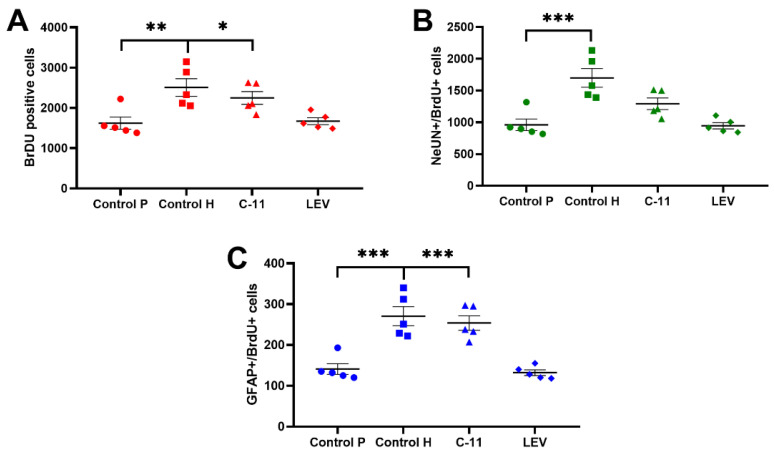
Evaluation of the neurogenesis process after the long-term administration of C11 and levetiracetam (LEV) in mice after pilocarpine (PILO)-induced status epilepticus (SE). (**A**)—The effect of long-term treatment with C11 and LEV on the total BrDU cells of PILO mice. (**B**)—The effect of long-term treatment with C11 and LEV on the newborn neurons of PILO mice. (**C**)—The effect of long-term treatment with C11 and LEV on the newborn astrocytes of PILO mice. The numbers of cells represent an estimate of the total number of positively labeled cells in the subgranular zone (SGZ) and granular cell layer (GCL) of the dentate gyrus (DG) of PILO mice in both hemispheres. The results were analyzed using one-way analysis of variance (ANOVA), followed by Dunnett’s test for multiple comparisons. Each bar represents the mean for five mice; error bars are S.E.M. (* *p* < 0.05, ** *p* < 0.01, and *** *p* < 0.001; *n* = 5).

The obtained results revealed clear differences between the PILO control and healthy control mice in the total number of bromodeoxyuridine (BrDU)-positive cells (1512 ± 152 vs. 2507 ± 217, respectively, *p* < 0.01; *n* = 5; Figure 2A) In this test, C11 significantly increased the total amount of BrDU-positive cells equal to 2106 ± 158. The LEV PILO group indicated similar levels of newborn cells when compared to PILO control mice (1614 ± 86.7 vs. 1518 ± 164, respectively, *n* = 5; Figure 2A).

#### 2.2.2. Evaluation of the Effects of Long-Term Administration of C11 and LEV on the Newborn Neurons in the Dentate SGZ and GCL of Mouse Hippocampus after PILO Induced SE

The obtained results showed significant differences in the amount of newborn neurons between the PILO control and healthy control mice (961 ± 90.6 vs. 1700 ± 147, respectively, *p* < 0.0001; *n* = 5; Figure 2B) Interestingly, an increase in the amount of neurons cells for C11 was observed in comparison to the PILO control mice (1292 ± 91.04 vs. 0.05961 ± 90.6, respectively, *n* = 5), although the difference was not statistically significant (Figure 2B).

#### 2.2.3. Evaluation of the Effects of Long-Term Administration of C11 and LEV on the Newborn Astrocytes in the Dentate SGZ and GCL of Mouse Hippocampus after PILO Induced SE

Differences in the level of newborn astrocytes were observed for the PILO control and healthy control mice. The average number of astrocytes for PILO control mice was 141.2 ± 13.3 (*p* < 0.0001; *n* = 5; Figure 2C) and, for the healthy control group, 270.8.4 ± 23.46 (*p* < 0.0001; *n* = 5), Additionally, a significant increase of newborn astrocytes was observed for C11 PILO mice (254 ± 17.9; *p* < 0.001; *n* = 5) when compared to the control PILO group (Figure 2C), whereas the level of astrocytes for LEV mice was similar to the PILO control group (132.2 ± 6.8, *n* = 5).

#### 2.2.4. Evaluation of the Effects of Long-Term Administration of C11 and LEV on Mouse Spatial Learning and Memory after PILO Induced SE

**Figure 3:**
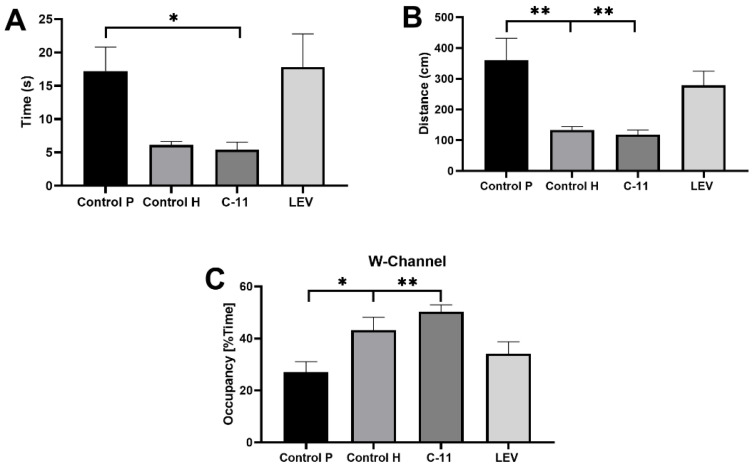
Evaluation of the long-term C11 and LEV administration on the escape latency (**A**), cumulative distance (**B**), and the mean percent of time spent in the W-Channel (**C**) in mice after PILO-induced SE in the Morris Water Maze test. Four groups were tested as follows: control PILO group, control healthy group, C11 (20 mg/kg), and LEV (10 mg/kg). The results were analyzed using one-way analysis of variance (ANOVA), followed by Dunnett’s test for multiple comparisons. Each bar represents the mean for eight mice; error bars are S.E.M. * *p* < 0.05 and ** p < 0.01; *n* = 8).

The results obtained from the Morris Water Maze test indicated a marked improvement in the process of spatial learning and memory in all treated mice in comparison to the control PILO group. All measured parameters in C11 mice: the mean escape latency (5.419 ± 1.123; *p* < 0.05; *n* = 7; Figure 3A, the mean distance (118.1 ± 15.24; *p* < 0.001; *n* = 7; Figure 3B), and mean percent of time spent in the W-Channel (50.34 ± 2.645; *p* < 0.001; *n* = 7; Figure 3C) averaged over the four quadrants were significantly more favorable compared to the PILO control group (17.21 ± 3589, 360.2 ± 72.18, and 27.12 ± 4.02, respectively, *n* = 7; Figure 3A–C) and quite similar to the healthy control group. Hence, statistically significant differences in the distance and W-Channel were also observed between PILO and healthy control mice. Surprisingly, LEV had no stimulating effect on the cognitive function of animals, and the obtained values of all three measured parameters did not differ significantly from the PILO control mice (Figure 3A–C).

#### 2.2.5. The Effect of Long-Term Treatment with C11 and LEV on the Level of NAA/Cr, GABA/Cr, Glc/Cr, Glt/Cr, and Gln/Cr in Mouse Brain after PILO-Induced SE

**Figure 4:**
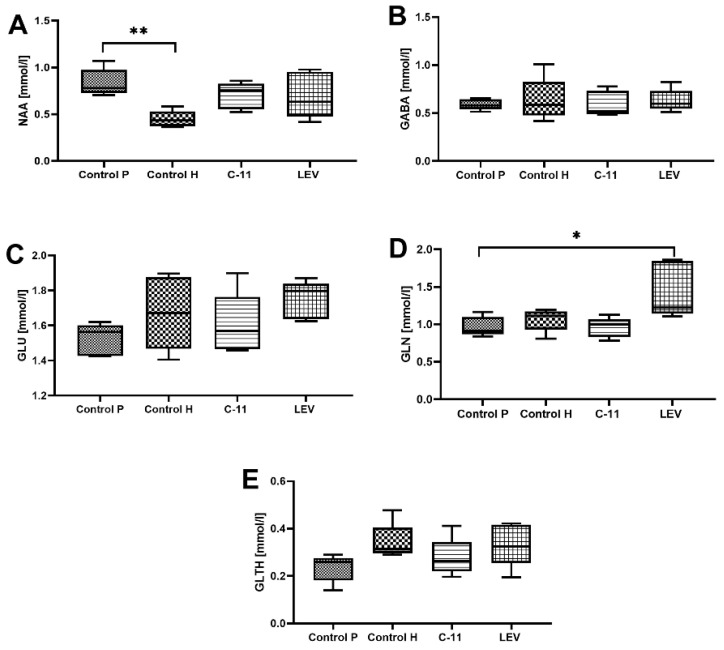
Evaluation of the long-term C11 and LEV administration on the level of (**A**) NAA; (**B**) GABA; (**C**) GLU; (**D**) GLN and (**E**) GLTH in mice of after PILO-induced SE. Experiments were performed on a 7T MRS scanner. Abbreviations: NAA—N-Acetyl Aspartate, GABA—Gamma-Amino Butyric Acid, GLU—Glucose, GLTH—Glutathione, GLN—Glutamine. The results were analyzed using one-way analysis of variance (ANOVA), followed by Dunnett’s test for multiple comparisons. Each bar represents the mean for five mice; error bars are S.E.M. * *p* < 0.05 and ** p < 0.01; *n* = 5).

The Magnetic Resonance Spectroscopy (MRS) results indicated no significant changes in the total amount of tested neurometabolites in both C11- and LEV (Figure 4A–E)-treated mice, except for the GLN level for LEV mice, where the significant increase of this metabolite was observed when compared to the PILO control group (1.441 ± 0.16 and 0.969 ± 0.05; *p* < 0.05; *n* = 5; Figure 4D). Interestingly, a statistically significant increase of the NAA/Cr level for the PILO control group was observed when compared to the healthy control mice (0.836 ± 0.060 and 448 ± 0.03, respectively; *p* < 0.001; *n* = 5; Figure 4A). It should be mentioned that the level of NAA/Cr for all treated PILO groups (C11 and LEV) was higher than the healthy control mice, but the difference was not statistically significant (Figure 4A).

## 3. Discussion

In the first part of the *in vitro* studies, the neuroprotective properties of our compound C11 were examined in human neurons and rat astrocytes under trophic stress and excitotoxicity conditions using the MTT test. This assay is a well-known and widely used assay for the evaluation of cell viability or proliferation; nevertheless, its basis is the measurement of mitochondrial dehydrogenase activity as an indicator of cell viability or proliferation. Due to that, in order to the proper interpretation of the obtained results before making the MTT assay, the cell responses to the investigated compounds were checked under the light microscope. The obtained results did not confirm our assumptions about C11’s ability to protect neurons; however, the results regarding the impact of C11 on the nerve cell viability under trophic stress conditions in astroglia cell culture indicated that C11 at concentrations 500, 1000, and 2500 ng/mL significantly induced the astrocytes viability. What is more, C11 also effectively increased the astrocytes amount in the standard conditions (complete medium with a standard amount of trophic agents). The obtained data may suggest the stimulating properties of C11 on the astrocytes viability, as well as the nutritional effect on astrocytes under trophic stress conditions, which the importance for neurodegeneration was earlier proved [28,29,30]. In the light of the data that astrocytes release several trophic factors [31], we suppose that the beneficial impact of C11 on the astrocytes viability in the serum deprivation medium was associated with the enhancement of both the metabolic activity and astrocytes number; of course, this hypothesis requires further verification. Considering the fact that neurotrophin production by astrocytes in response to brain tissue injury is a well-described mechanism of neuroprotection [32,33], examination of the C11 impact on these processes seems to be reasonable.

Although reactive astrocytes have been mainly regarded as detrimental for repair, recently, there have been reports that they are able to promote neurorestorative processes [34,35]. Thus, C11 is worth studying towards neuroprotective efficacy against a variety of neurological disorders in which neurodegenerative or neuroinflammatory processes are involved.

Data from *in vivo* studies assessing the potential changes in the process of neurogenesis after C11 treatment in a model of PILO SE in mice showed the stimulating properties of C11 on stem cell proliferation when compared to the PILO control mice, where the level of newborn BrDU-positive cells was significantly lower. However, it should be mentioned that the relatively long time between PILO-SE and the quantification of neurogenesis may also have an impact on the increase of the newborn cells. Typically, neurogenesis has been shown to increase within several days after PILO-SE in animals, but after several weeks, when the spontaneous recurrent seizures occur, a significant decreased is observed [36]. Evaluation of the potent changes in neurogenesis several weeks after PILO-SE enables a thorough analysis of the newly formed cells in the epileptic brain [37]. Our recent study using the long-term administration of C11 in healthy mice did not indicate any disturbances in the hippocampal neurogenesis, which was also confirmed in the present study [27]. Interestingly, a long-term treatment with C11 of PILO mice significantly increased the total amount of newborn cells, including astrocytes, similarly to the level of the healthy control group. For newborn neurons, we also observed an increase of cells most close to the level of neurons in healthy animals; however, the difference with PILO control mice neurons was not statistically significant. It should be noted that the results from our previous studies on healthy mice indicated the neutral impact of chronic administration of C11 on astrocytes when compared to the control group [27]. On the other hand, LEV, despite its unique anticonvulsant mechanism of action in SE [38] and neuroprotective properties [39,40], turned out to be ineffective in improving the neurogenesis (including both neurons and astrocytes) of PILO animals. Similarly, a lack of LEV efficacy in a PILO-induced model of epilepsy was shown by Zagaja et al. [18]. Moreover, in our previous studies, LEV (10 mg/kg) also decreased the level of newborn neurons in healthy mice [27]. In contrast, Yan et al. [41] indicated that the long-term treatment with LEV at high doses (300 and 600 mg/kg) enhanced the cell proliferation and neuronal differentiation in the hippocampal DG of mice. Interestingly, Ithoh and coworkers [42] showed that two days of treatment with LEV (360 mg/kg) after PILO-induced SE in mice suppressed neuroinflammation and spontaneous recurrent seizures. In turn, a recent study by Vyas et al. [43] indicated the partial protective activity of seven-day prophylactic treatment with LEV at dose of 200 mg/kg but not for sodium valproate (VPA; 300 mg/kg) and carbamazepine (CBZ; 100 mg/kg) in mouse lipopolysaccharide (LPS)-primed + PILO-induced SE. One important thing to note regarding the above-mentioned studies of the anticonvulsant and neuroprotective effect of LEV in animal PILO-induced SE is its dose. In our study, LEV was administered chronically for 10 days at a dose of 10 mg/kg starting 72 h after SE induction. The LEV dose was selected based on our previous studies [13,27], but we also took into account the relatively low dose of C11 (20 mg/kg). It was reasonable to choose similar doses for both the aforementioned anticonvulsants. Therefore, it should be assumed that the lack of LEV neuroprotective activity may result from the relatively low dose (10 mg/kg) used in the studies.

The results showing the reduction of astrocytes in the mouse dentate gyrus after PILO-induced SE were also shown by Borges and coworkers [44], although selective astrocyte death in the dentate hilus after PILO SE was dependent on the species and method used to induce SE. A significant degeneration was observed up to three days after PILO-SE, followed by a gradual increase in the number of GPAP-positive cells. In our study, a BrDU proliferation marker was injected 7 days after PILO-SE, and still, the level of GFAP cells remained significantly lower compared to healthy control mice. The opposite results were presented by Zhang et al. [45] studying the anticonvulsant effect of baldrinal in mouse PILO-SE. Seventy-two hours after SE, a high increase of GFAP-positive cells was noted, which can be explained by the fact that, in animal models of epilepsy, astrocytes are rapidly activated with the hypertrophy of cell bodies, thus increasing the expression of GFAP [46]. Astrocytes are known to be the most important neural cell type for the maintenance of brain homeostasis and to cooperate with neurons on many levels. What is of great importance is the time after which the qualitative, as well as quantitative, analyses of newly formed NEuN or GFAP cells after SE induction are performed.

The *in vivo* assessment of neurogenesis using MRS showed no statistically significant differences in the level of several selected neurometabolites important for neurogenesis in C11 mice when compared to the PILO control group. However, all PILO groups showed an increased level of NAA/Cr, although only for PILO control mice was the difference significant. MRS enables the identification and quantification of the levels of the brain metabolites, such as NAA and glutathione and levels of the neurotransmitters, such as GLU, GLN, and GABA, which may be relevant to epileptogenesis [47]. We can find many scientific data from animal models of epilepsy (especially post-SE) showing a significant decrease of NAA [47,48,49]. An increased level of NAA was already reported in our previous study [27] in lacosamide (LCM)- and LEV-treated mice. Based on the previous research methodology, MRS was performed three weeks after the last anticonvulsant injections and five weeks after PILO-SE. It is surprising that all PILO groups (control, C11, and LEV) 5 weeks after SE induction maintained elevated NAA levels. One of the rational explanations for the difference between quantitative reduced neurogenesis and MRS seems to be a different time point of detection.

Disturbed cognitive functions after PILO-SE in mice were observed in the Morris Water Maze (MWM) test, which is one of the most common tests for examining spatial learning and memory in mice. Bearing in mind that PILO-induced SE in mice is responsible for learning and memory dysfunctions [50,51,52,53,54], we focused on three of the most important parameters; time and distance to reach the platform and the average time spent in W-Channel. The obtained results showed a significant beneficial effect of the long-term administration of C11 in PILO mice in comparison to the PILO control and LEV groups. As we showed in our previous studies, the chronic administration of C11 in healthy mice did not impair learning and memory in the test animals [27]. Moreover, in the current research, we showed that C11 significantly shortened the time within which the PILO animals reached the platform. The time to reach the platform was also very similar to the animals from the healthy control group, although not statistically significant. Additionally, C11 mice similar to the healthy control group turned out to have a significantly better percent of time spent in the W-Channel, which clearly indicated a lack of spatial orientation disturbances in the tested animals. A proper neurogenesis and undisturbed cognitive functions after PILO SE in mice indicated a neuroprotective effect of C11, although its mechanism of action remains not fully defined.

Summing up, C11 was proven to significantly stimulate the proliferation of newborn cells, as well as their migration and differentiation into neurons and astrocytes, as well as protect the cognitive functions in the mouse PILO-induced SE model. To explain all the beneficial properties of C11, its anticonvulsant and neuroprotective mechanism of action should be identified, which, according to our data, seems to be multidirectional. *In vitro* radioligand-binding studies revealed the binding affinity of C11 towards the L-type Ca^2+^ channels and Na^+^ channels (Site 2), which might contribute to its antiepileptogenic effects [22]. Further *in vitro* investigations using patch–clamp experiments in rat prefrontal cortex pyramidal neurons determined the influence of C11 on fast voltage-gated sodium channels. The *in vivo* research done so far using the mouse PTZ kindling model of epilepsy indicated that several distinct GABA-mediated mechanisms might be responsible for the protective effect of C11, including changes in GABA release, modulation of GABA transaminase activity, and changes in the expression of GABA transporters and/or GABAA receptor subunits [55]. Therefore, both the *in vitro* and *in vivo* data obtained so far certainly allow for the presumption that the anticonvulsive and neuroprotective effectiveness of C11 is caused by the involvement of various mechanisms of action.

Looking for a new potent anticonvulsant drug candidate that would simultaneously protect neurons and cognitive functions in refractory epilepsy with a tendency towards SE is a priority and a challenge for researchers. Bearing in mind our *in vitro* results confirming a protective effect of C11 on astrocytes under excitotoxicity or trophic deprivation-mediated neuronal death, as well as its beneficial effect on neurogenesis and cognitive functions, more advanced molecular research is certainly worthwhile to determine the exact neuroprotective mechanism of action. Moreover, further preclinical studies on the neuroprotective properties of C11 could open up new frontiers of research for this substance as a potential drug candidate in other neurodegenerative diseases.

## 4. Materials and Methods

### 4.1. In Vitro Study

#### 4.1.1. Reagents

All reagents and kits were purchased from Sigma-Aldrich (St Louis, MO, USA), unless otherwise indicated.

#### 4.1.2. Neuroblastoma Cell Line

Human neuroblastoma SH-SY5Y cells were obtained from ECACC (European Collection of Cell Cultures, Salisbury, UK) and cultured according to its recommendations.

#### 4.1.3. Differentiation of SH-SY5Y towards Neuronal Cells

SH-SY5Y cells’ differentiation to the neuronal cells was performed according to the previously described method, but the experiments were conducted on neuronal cells cultured for 12 days [56].

#### 4.1.4. Astroglia Cell Culture

Astroglia cell culture was prepared from cortices of 3-dayold newborn Wistar rats. The tissue was dissociated with a 0.25% trypsin-EDTA solution. Obtained cell suspension at a density of 1 × 10^6^ cells/mL was resuspended in Dulbecco’s Modified Eagle Medium/Nutrient Mixture F-12 (DMEM/F12) medium supplemented with 10% fetal bovine serum (FBS), penicillin (100 U/mL), and streptomycin (100 mg/mL). The cell suspension was maintained in a humidified atmosphere of 95% air and 5% CO_2_ at 37 °C (standard conditions). The culture medium was changed daily until the culture reached the confluence (10 days); then, culture vessels with growing cells were shaken overnight in an orbital shaker at 210 rpm in order to remove the fewer adherent cells (neurons, microglia, and oligodendroglia). Following this shaking procedure, the culture became enriched with flat cells displaying typical astrocyte morphology. Immunostaining with a primary antibody for glial fibrillary acidic protein (GFAP) (polyclonal, DAKO, Glostrup, Denmark) revealed that astrocytes accounted for around 95% of the cells in the culture.

#### 4.1.5. Cell Viability Assessment—MTT Assay

Both neurons and astrocytes were exposed to serial dilutions of C11 (100, 500, 1000, and 2500 ng/mL) used alone or in combination with 3-mM glutamate. Solutions of the investigated compound were prepared in the culture medium suitable for given cells (as described above). In the case of experiments performed in conditions of trophic stress, solutions of C11 were prepared in the medium deprived of B-27 supplement (neurons) or the medium with reduced to 2% of FBS (astrocytes). Metabolic activity of nerve cells in the response to C11 was examined after 48 h of treatment using the MTT assay. In order to properly interpret the obtained results before the addition of MTT, all plates were checked under the light microscope, and afterward, the MTT solution (5 mg/mL in phosphate-buffered saline, PBS) was added for 3 h. Resultant crystals were solubilized overnight in SDS buffer pH 7.4 (10% SDS in 0.01 N HCl) and the product quantified spectrophotometrically by measuring the absorbance at a 570-nm wavelength using a microplate reader (BioTek ELx800, Highland Park, Winooski, VT, USA). The results were presented as a percentage of cell viability treated with the investigated compounds versus cells grown in the control medium (indicated as 100%).

### 4.2. In Vivo Study

#### 4.2.1. Animals and Experimental Conditions

All experiments were performed on 6-week-old male CB57/BL (20–22 g) mice kept in colony cages with free access to food and tap water ad libitum, under standardized housing conditions (natural light-dark cycle, temperature 21 ± 1 °C). After 7 days of adaptation to laboratory conditions, the animals were randomly assigned to four experimental groups consisting of eight mice. For the MRS and quantitative analysis of neurogenesis, five from eight mice were analyzed. Experimental procedures related to the care of animals and protocols used in the study were approved by the Local Ethics Committee at the University of Life Science in Lublin (No 35/2016).

#### 4.2.2. Status Epilepticus (SE) in Mice

Mice were administered an intraperitoneal (i.p.) injection of methylscopolamine (1 mg/kg) dissolved in water 15–30 min prior to injection of PILO to reduce the peripheral cholinergic effects of PILO. Experimental animals were then injected i.p. with a single dose of PILO 300 mg/kg. Control mice were age-matched with treated mice and administered a comparable volume of vehicle after the initial methylscopolamine treatment. Mice were carefully observed after PILO injection to catch first symptoms of convulsions. Seizure behavior occurred approximately 15 min after the PILO injection. The category and the number of generalized convulsive seizures in each 1/2-h period were tallied. A modified version of the seizure scale described by Racine and coworkers [57] with categories 1–5 were used to identify the seizure severity. Categories one and two (i.e., facial automatisms, tail stiffening, and wet dog shakes) were considered as a group to avoid subjectivity in assessing the seizures. Category 3, 4, and 5 were considered to be generalized, convulsive seizures. After 2 h of observation, animals were injected with diazepam (1 mg/kg) to stop SE. Animals with category 4 and 5 seizures that survived SE became candidates for the next step of the experiment. For the animals with no seizures, euthanasia with carbon dioxide inhalation was performed.

#### 4.2.3. Drugs

The following drugs were used in this project: LEV (Keppra; UCB Pharma, Brussels, Belgium), 5-bromo-2′-deoxyuridine BrDU (Sigma Aldrich, St. Louis, MO, USA), diazepam (Relanium, GSK, London, UK), and pilocarpine and scopolamine (Sigma Aldrich, St. Louis, MO, USA). C11 compound was synthetized in the Department of Medicinal Chemistry, Jagiellonian University Medical College (Krakow, Poland) according to the procedure described previously [22]. All substances were suspended in a 1% solution of Tween 80 (Sigma, St. Louis, MO, USA) in water for injection (Baxter, Poland), All drugs were injected intraperitoneally (i.p.) with 1-mL syringes as a single injection in a volume of 0.005 mL/g.

#### 4.2.4. Drugs Administration

Animals were divided into 4 groups (8 mice per group):

1. PILO C11,
2. PILO LEV,
3. PILO Control group (PILO + water for injections + Tween 80), and
4. Healthy Control group (water for injections + Tween 80)

**Figure 5:**
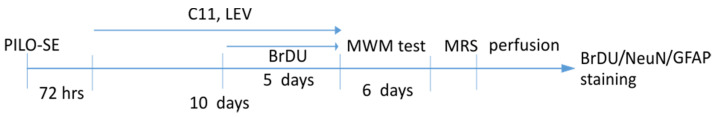
Experimental study design.

Animals were administered with C11, LEV, and water for injections + Tween 80 72 h after SE induction once a day for the subsequent 10 days. Fresh drug solutions were prepared ex tempore each day of the experiment. LEV was administrated intraperitoneally (i.p.) at dose of 10 mg/kg based upon information about their efficacy in the experimental models of epilepsy found in the literature [10,18]. C11 was injected at a dose of 20 mg/kg, according to the quantitative pharmacological parameter effective dose (ED_50_) from the 6-Hz test [22]. Additionally, BrDU (a marker of cell proliferation) was given as one more single injection for the last 5 days of the treatment. Animals were subjected to transcardial perfusion 3 weeks after the last BrDU injection. The experimental design used in the study is shown in Figure 5.

#### 4.2.5. Behavioral Study-Spatial Learning and Memory (MWM Test)

Animals underwent a behavioral test 24 h after the last anticonvulsant injection according to the methods described earlier [27]. There was one daily session consisting of four 60-s trials (each trial form a different quadrant of the pool) for 5 consecutive days. Twenty-four hours after 5 days of training, the final test (probe test) was performed. Three parameters were measured: escape latency, distance, and time spent in the W-Channel. Obtained results were analyzed based on the average values of the parameters tested from all quadrants for each animal in the group.

#### 4.2.6. Magnetic Resonance Spectroscopy (MRS) 

Three weeks after the last anticonvulsant injection, 5 animals from each experimental group were subjected to MRS to obtain more information about any neurodegenerative changes in the mouse brain. Proton Magnetic Resonance Spectroscopy (^1^HMRS) acquisition, as well as spectra processing, was described in detail in our previous publication [27].

According to the experimental data, a neurochemical profile of about 17 metabolites can be quantified using MRS in mice brains, including alanine (Ala), aspartate (Asp), creatinine (Cr), aminobutyric acid (GABA), glucose (Glc), glutamate (Glu), glutamine (Gln), glutathione (GSH), glycerophosphorylcholine (GPC), phosphorylcholine (PCho), myo-inositol (Ins), lactate (Lac), Nacetylaspartate (NAA), N-acetylaspartylglutamate (NAAG), phosphocreatine (PCr), scyllo-inositol (Scyllo), and taurine (Tau) [58,59]. A creatinine (Cr) was used as a criterion to quantitatively analyze alterations of the other metabolites [60]. Thus, the ratio of several selected neurometabolite/Cr clinically important for neurogenesis was evaluated: NAA, GABA, Glc, Glth, and Gln.

#### 4.2.7. Brain Slice Preparation

For determining the influence of C11 and LEV on the proliferation, migration, and differentiation, 3 weeks after last anticonvulsant and BrDU injections, mice were anesthetized with isoflurane anesthesia with a premedication of analgesic drugs and perfused with ice-cold saline followed by freshly prepared, ice-cold 4% paraformaldehyde and then processed according to the methods described earlier [10,14,27].

#### 4.2.8. Immunohistochemical Staining-Neurogenesis

Fifty-micrometer sections were stored at 4 °C in cryoprotectant until needed. Free-floating sections were immunostained according to the methods described previously [10,14,27].

#### 4.2.9. Confocal Microscopy and Cell Counting

Confocal imaging was performed using a Nikon A1R confocal system microscope (Tokyo, Japan). Quantitative analysis of newborn BrDU cells colocalizing with NeUN and GFAP cells included the GCL and SGZ of the mouse DG using the methods described in our previous studies [10,14,18,27].

### 4.3. Statistical Analysis

For the *in vitro* study, data was presented as the mean value and standard error of the mean (SEM) according to the previous study [56].

Results from *in vivo* study were analyzed using one-way analysis of variance (ANOVA), followed by the Dunnett’s test for multiple comparisons, and performed using commercially available GraphPad Prism version 4.0 for Windows (GraphPad Software, San Diego, CA, USA).
